# Insights into the complexation of *N*-Allyl-4-(4-(*N*-phenylureido)benzylamino)-1,8-naphthalimide with various anions

**DOI:** 10.1038/s41598-017-02470-0

**Published:** 2017-05-31

**Authors:** Andrew J. Blok, Martin R. Johnston, Claire E. Lenehan

**Affiliations:** 1 School of Chemical and Physical Sciences, Flinders University, Sturt Road, Bedford Park, South Australia Australia; 2 Flinders Centre for NanoScale Science and Technology, Flinders University, Sturt Road, Bedford Park, South Australia Australia

## Abstract

A new urea functionalised 4-amino-1,8-naphthalimide based fluorescent anion sensor was synthesised in 64% yield over three steps. Fluorescence and ^1^H NMR titrations showed that the sensor complexes strongly with acetate and dihydrogen phosphate and to a lesser extent bromide. The corresponding binding stoichiometries were examined using ^1^H NMR titrations. Results show that the sensor molecule initially forms 1:1 complexes through hydrogen bonding to the urea moiety, followed by secondary complexation to form higher order host:guest stoichiometries. Specifically, oxyanions complex to the sensor via hydrogen bonding through synergistic aryl C-H and N-H anion interactions in a 1:2 sensor:oxyanion arrangement. Furthermore, 2:1 sensor:oxyanion complexes are formed through an oxyanion linkage between two urea functionalities on different host molecules. This contrasts the majority of previous reports for similar hosts, which indicate 1:1 binding stoichiometry.

## Introduction

It has been well established that molecules based on the 4-amino-1,8-naphthalimide fluorophore in combination with urea and thiourea anion recognition groups, are potent “on-off” sensors for the dihydrogen phosphate, acetate and fluoride anions^1–8^. In this sensor motif the anion binds directly to the urea (or thiourea) recognition group on the sensor. This results in a decrease in fluorescence intensity due to photoinduced electron transfer (PET) from the receptor to the fluorophore^1^. Whilst, sensors incorporating 4-amino-1,8-naphthalimide are typically reported to bind with 1:1 stoichiometry through the urea moiety, it has also been reported that anion binding to a urea may cause a “positive allosteric effect” that facilitates a secondary binding event^9^. For example, dos Santos *et al*. reported the activation of an amide binding site towards anions resulting from an inductive effect due to primary anion binding at the urea site. Overall, this resulted in a 1:2 (sensor:anion) binding stoichiometry. In addition to this potential second binding event, it has been reported that the inherent dipole of the naphthalimide moiety can generate polarised aryl C-H donors which may be used for cooperative hydrogen bonding interactions with anionic guests^10^. Theoretical calculations have also shown that C-H bonds can exhibit binding affinity^11–13^, with in some cases C-H bonds showing up to 50% of the strength of O-H and N-H groups^12^.

**Figure 1:**
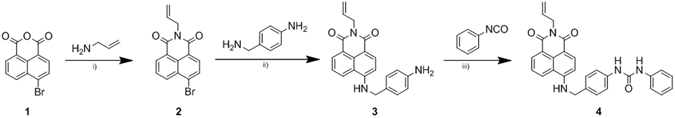
Synthesis of *N*-allyl-4-(4-(*N*-phenylureido)benzylamino)-1,8-naphthalimide. i) EtOH, reflux, 3 h, 94% ii) 110 °C, 2 h, 72% for 3, iii) DMF, TEA, r.t, 15–84 h, **4** 94%.

We report here the development of an anion sensor using urea as the complexing moiety and 4-amino-1,8-naphthalimide as the optical probe. We show that the new sensor (**4**, Fig. 1) forms both 1:1 and higher order complexes with acetate and dihydrogen phosphate oxyanions in hydrated (0.5% water) DMSO solution (as evidenced by ^1^H NMR spectroscopy). Binding of the oxyanions to the urea receptor on **4** was found to activate a second binding site, resulting in a 1:2 sensor:anion complex. This second site was only activated after the first binding event. In contrast to dos Santos *et al*.^9^, the 4-amino-1,8-naphthalimide sensor presented in this report does not contain a second amide site, nor is it conjugated to the urea binding site. We proposed that the 4-amino NH group in **4** may act in cooperation with a polarised naphthalimide C-H group to form a second binding site^10^. Thorough analysis of the ^1^H NMR binding isotherms along with molecular modelling support the hypothesis that the second anion is bound through an aryl-C-H- at the C5 position in concert with the adjacent N-H on the C4 position. In addition to this, 2:1 sensor:oxyanion complexes were observed to occur, whereby the anion formed a bridge between two urea recognition groups on different host molecules. This is the first report where these three binding stoichiometries have been observed for the 4-amino-1,8-naphthalimide based sensors.

## Results and Discussion

Sensor **4** was prepared in three steps according to the synthetic route in Fig. 1. The imide **2** was synthesised as described by Niu *et al*.^14^. Here the anhydride **1**, was reacted with allylamine in ethanol under reflux conditions and allowed to cool yielding **2** as an off white powder in 94% yield. Subsequent nucleophillic aromatic substitution of **2** with *p*-aminobenzylamine resulted in **3**, which was collected as a mustard yellow powder after precipitation upon the addition of water to the reaction mixture. The yield after recrystallisation from hot ethanol was 72%. Finally, excess phenyl isocyanate was reacted with **3** in the presence of triethylamine to form the bright yellow sensor molecule, **4** in 94% yield.

**Figure 2:**
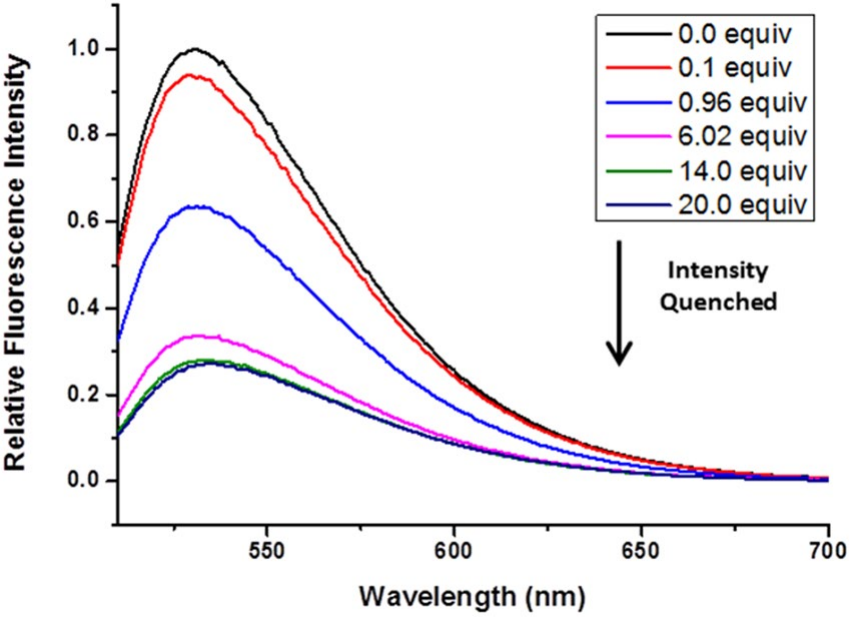
Fluorescence spectra of **4** with increasing equivalents of tetrabutylammonium acetate in hydrated (0.5% v/v) DMSO, λ_ex_ = 503.93 nm.

The affinity of **4** for the acetate, dihydrogen phosphate, and bromide anions (all used as their tetrabutylammonium salts) was evaluated using fluorescence spectrophotometry and ^1^H NMR spectroscopic titrations. The affinity of **4** with fluoride was also investigated, however as per previous reports^1^ was found to deprotonate the 4-amino NH proton, complicating the binding analysis further. Therefore the interaction with fluoride will not be dealt with in this paper. All titrations were performed in DMSO-d6 with 0.5% v/v water added to reduce errors due to water absorption from the atmosphere by the highly hygroscopic DMSO^15^. Similar to previous reports examining the affinity of naphthalimide sensors for anions^6^, the addition of acetate to **4** resulted in significant quenching of the fluorescence emission, with approximately 27% of the original signal remaining after the addition of 20 equivalents (Fig. 2).

**Figure 3:**
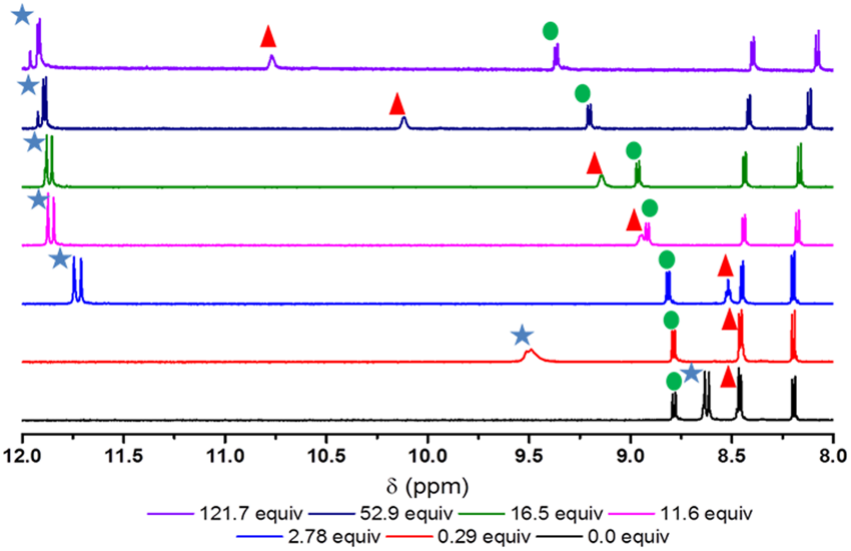
^1^H NMR spectra (600 MHz, 6.24 mM, 293 K) of **4** with increasing amount of tetrabutylammonium acetate added in hydrated DMSO-d6 (0.5% v/v). The blue star indicates the signal arising from the urea N-H protons, the green circle the signal arising for the C5 naphthalimide proton and the red triangle the 4-amino NH proton.

The intermolecular interactions between **4** and acetate were further examined using ^1^H NMR spectroscopy to gain an understanding of the complexation geometry. Fitting of ^1^H NMR spectral changes can be achieved by local and global optimisation methods and there are a range of software programs available for this purpose^16–21^. This research used the commercially available HypNMR as it is suitable for fast exchange and is not limited by number of binding constants or nuclei^22^. As shown, (Fig. 3) a downfield shift of approximately 3.3 ppm was evident for both urea N-H proton resonances, consistent with strong H-bonding between the urea N-H and the acetate anion. Similarly the aromatic C-H adjacent to the urea receptor, had a measurable change in its chemical shift, albeit somewhat smaller (~0.1 ppm) with increasing acetate concentration. The resonance assigned to the 4-amino proton, on the other hand, barely moved (~0.01 ppm) prior to the addition of one equivalent of acetate, after which a significant downfield shift was observed (~2.3 ppm). This trend was mirrored by the C5 naphthalimide proton resonance which had moved ~0.6 ppm downfield after the addition of ~120 equivalents of acetate. The remaining naphthalimide proton resonances had similar, but less significant shifts (<0.16 ppm).

These observed shifts are inconsistent with a purely 1:1 (sensor:acetate) binding interaction as reported for similar molecules^5^, and indicate a more complex, stepwise, binding process (see Supplementary Figure S1 for the best fit achieved using a 1:1 binding model). The downfield shifts in the ^1^H NMR titration for the 4-amino and the C5 proton resonances are consistent with **4** binding to a second acetate anion in a coordinated manner. It has been reported that anion binding to a urea moiety may cause a “positive allosteric effect” resulting in a secondary binding event^9^. However, in that case, an amide group was directly conjugated to the urea *via* a phenyl group. In this work, the amine is not conjugated to the urea and is separated from the recognition unit *via* a methylene spacer. Alternately, MacDonald showed that the inherent dipole of the naphthalimide moiety can generate polarised C-H donors for cooperative hydrogen bonding interactions with anionic guests^10^, and is likely to be the cause of the secondary binding event. As mentioned earlier, the ability for the C-H bond to be involved in anion binding has also been supported by theoretical studies^11, 13, 23^.

**Figure 4:**
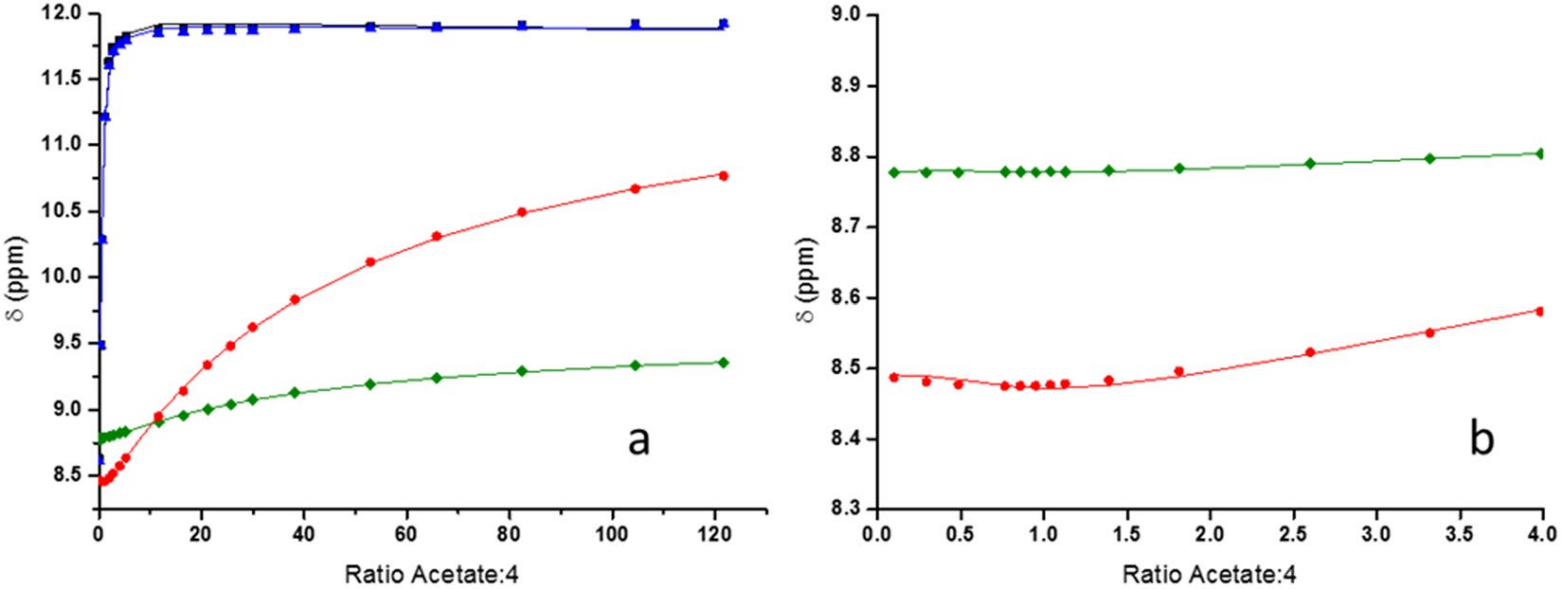
(**a**) ^1^HNMR (600 MHz) binding curves for urea (black square and blue circle), C5 naphthalimide (green diamond) and 4-amino NH proton resonances (red circle), with the fits calculated using a 2:1, 1:1 and 2:1 binding model in HypNMR for complexes formed between **4** and tetrabutylammonnium acetate represented by the coloured lines. (**b**) An expansion of Fig. 4a showing the change in chemical shift of the C5 naphthalimide and 4-amino NH protons.

Global fitting of a stepwise 1:1 and 1:2 (**4**:acetate) model to the ^1^H NMR data, using HypNMR, was unable to be refined. It is likely that, when in the presence of a significant excess of **4**, the acetate could interact with more than one molecule of **4**. Here the urea receptors of two adjacent sensor molecules could self-assemble to trap an acetate anion between them in a similar manner to the pre-organised complex with two thioureas as reported by Pfeffer^24^. A binding model involving the initial formation of a 1:1 (**4**:acetate) complex, followed by the subsequent formation of 2:1 and 1:2 (**4**:acetate, Equations 1–3), complexes closely fitted the experimental data and was able to be refined. The respective log *K* values (Table 1) were found to be log *K*_*11*_ = 3.17 ± 0.02, log *K*_*21*_ = 2.38 ± 0.009 and log *K*_*12*_ = 0.51 ± 0.04. The resulting calculated shifts for the urea and 4-amino proton resonances are shown overlaid with the raw data in Fig. 4(a and b).

1 $${\rm{4}}+{\rm{AcO}}{}^{-}\leftrightarrow {[4:\mathrm{AcO}]}^{-},{K}_{{\rm{11}}}[4][{{\rm{AcO}}}^{-}]={[4:{\rm{AcO}}]}^{-}$$

2 $${\rm{4}}+{[4:\mathrm{AcO}]}^{-}\leftrightarrow {[{4}_{2}:\mathrm{AcO}]}^{-},\,{K}_{{\rm{21}}}[4][4:{{\rm{AcO}}}^{-}]={[{4}_{2}:{\rm{AcO}}]}^{-}$$

3 $${{\rm{AcO}}}^{-}+{[4:\mathrm{AcO}]}^{-}\leftrightarrow {[4:{(\mathrm{AcO})}_{2}]}^{2-},{K}_{{\rm{12}}}[{{\rm{AcO}}}^{-}][4:{{\rm{AcO}}}^{-}]={[4:{(\mathrm{AcO})}_{2}]}^{2-}$$

**Table 1:** Binding constants for the interaction between **4** and an anion as determined by ^1^H NMR spectroscopy using HypNMR software.

Anion	log *k*_*11*_	log *K*_*21*_	log *K*_*12*_
Acetate	3.17 ± 0.02	2.38 ± 0.009	0.51 ± 0.04
Dihydrogen Phosphate	3.668 ± 0.009	3.028 ± 0.002	0.18 ± 0.12

Equations 1–3. Binding model used for fitting the ^1^H NMR data involving the initial formation of a 1:1 complex followed by subsequent formation of 1:2 and 2:1 complexes. AcO^−^ represents acetate.

**Figure 5:**
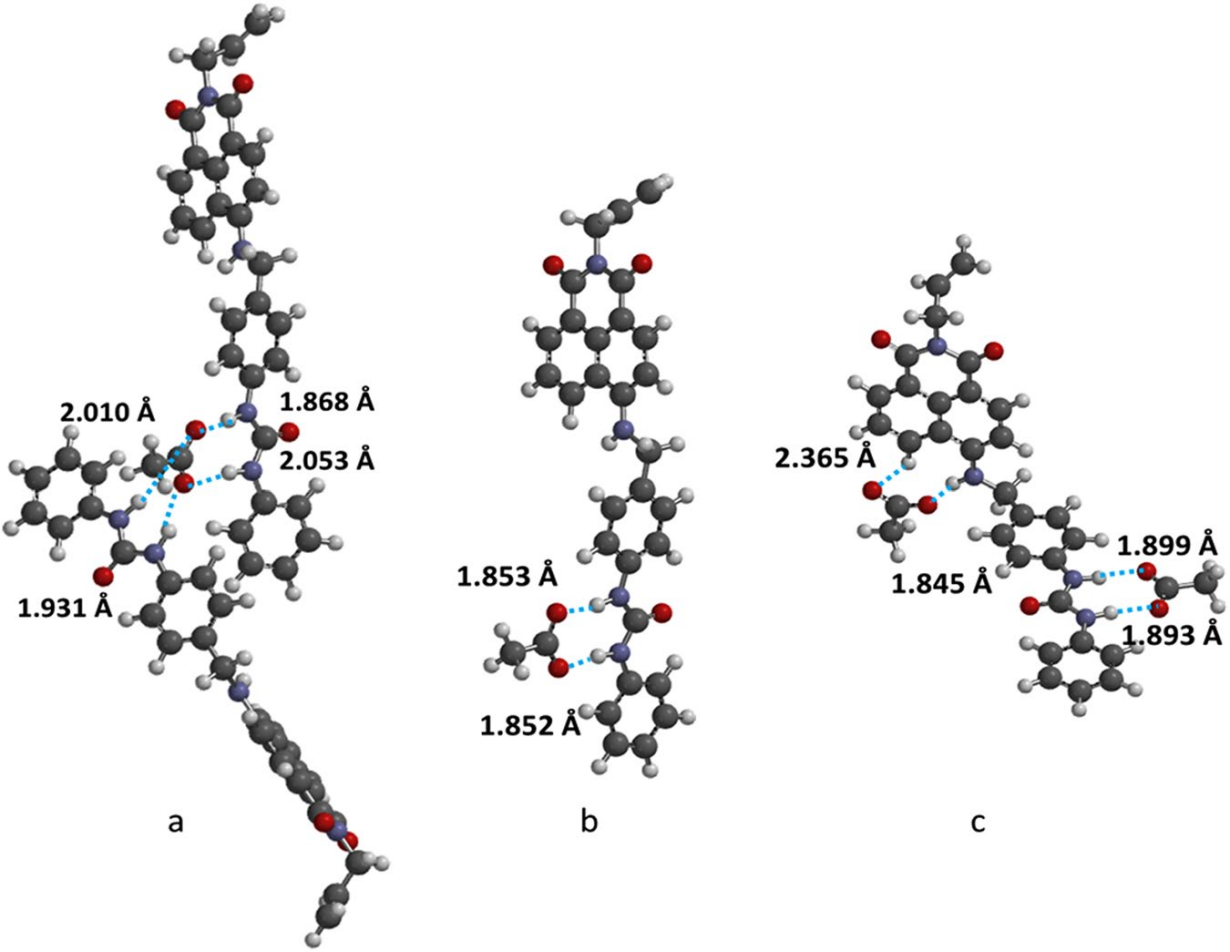
(**a**) 2:1, (**b**) 1:1 and (**c**) 1:2 Proposed structure of adducts formed between **4** and acetate showing H–bonded pairs and bond lengths (Hartree Fock 6–31G*).

1:1 and 2:1 (**4**:acetate) complexation occurs through the urea moiety, as evidenced by the large downfield shifts of the urea protons. The binding of a second acetate ion to the 1:1 complex is evidenced by the downfield shifts of the N-H and naphthalimide proton resonances after 1 equivalent of acetate was added. This second complexation occurs with a log *K* value of an order of magnitude lower than for the 1:1 complex (0.58 v 3.17). This would be expected, as the addition of a second anion to an already negatively charged complex would be unfavourable. In addition, this site is only activated after the primary binding event. Further insight into the geometry of the complexes was gained using molecular modelling (Spartan 10™, Version 1.1.0, Wavefunction Inc.). Geometry optimisations were undertaken at *ab-initio* (Hartree Fock) level using a 6–31G* basis set. Optimised structures for the 2:1, 1:1 and 1:2 **4**:acetate complexes are shown in Fig. 5(a,b and c). The geometry of the 1:1 complex (Fig. 5b) shows complexation between the urea moiety and the acetate anion, in a similar manner to previously reported^5^. Here the H-bond distances between the urea protons and the acetate ion were calculated to be 1.853 and 1.852 Å, respectively.

Modelling the addition of a second anion resulted in a 1:2 (**4**:acetate) complex, with the second acetate hydrogen bonding to the 4-amino and C5 naphthalimide protons. The resulting complex is shown in Fig. 5c. The positioning of the second acetate is consistent with binding to the 4-amino and C5 naphthalimide protons, with bond distances of 1.845 and 2.338 Å, respectively. This is consistent with our NMR titration results indicating that this second interaction is weaker than the initial 1:1 binding, as reflected by the log *K* values.

Modelling of the 2:1 (**4**:acetate) complex resulted in the geometry shown in Fig. 5a. Here, the acetate anion is positioned between two urea moieties on different hosts, with an average hydrogen bond length of approx. 1.96 Å, indicating a relatively strong interaction, mirroring the results from the NMR titration.

The interaction of dihydrogen phosphate with **4** was very similar to that of acetate (^1^H NMR spectra is in Supplementary Figure S2). The fluorescence of **4** was quenched with approximately 30% of the original signal remaining after the addition of 20 equivalents of anion (Supplementary Figure S3), only slightly less quenching than that of acetate. Similarly to acetate, fitting of the ^1^HNMR titration data of **4** with phosphate was consistent with the formation of 2:1, 1:1 and 1:2 (**4**:dihydrogen phosphate) binding stoichiometries (Supplementary Figure S4a and b). Large downfield shifts (~2.1 ppm) were again evident for both urea proton resonances, consistent with intermolecular interactions between the urea receptor and the dihydrogen phosphate anion. The position of the 4-amino and C5 protons shifted downfield (~0.85 ppm and ~0.25 ppm, respectively) only after the addition of one equivalent indicating a second complexation at the 4-amino site. The extent of the shifts of the 4-amino and C5 naphthalimide proton, were smaller than those for acetate, indicating a weaker interaction. Fitting of a stepwise model, incorporating the 1:1, 2:1 and 1:2 (**4**:dihydrogen phosphate) complexes resulted in log *K*_11_ = 3.668 ± 0.009, log *K*_21_ = 3.028 ± 0.002 and log *K*_12_ = 0.18 ± 0.12 (Table 1, Supplementary Figure S4a and b). Proposed geometry optimised (HF-6–31G*) structures for the 2:1, 1:1 and 1:2 (**4**:dihydrogen phosphate) complexes are shown in Supplementary Figure S5.

In contrast to acetate and phosphate, the addition of the large spherical bromide ion to **4** resulted in minimal quenching of fluorescence intensity (15% decrease after 20 equivalents, Supplementary Figure S6). Similarly, a ^1^H NMR titration of **4** with bromide showed evidence of weak interactions with the urea proton resonances both shifting downfield ~0.74 ppm (Supplementary Figure S7). The 4-amino and C5 naphthalimide proton peaks exhibited downfield shifts of 0.36 and 0.26 ppm, respectively. A weaker interaction is consistent with reports for a similar molecule^5^, as the large size and low charge density of the anion are likely to decrease binding strength. In this case a 1:1 binding stoichiometry fitted the experimental data, and there is limited evidence of any secondary binding events (Supplementary Figure S8). The proposed geometry optimised structure of the 1:1 complex reflected the weak interaction, with bond lengths of 2.496 and 2.571 Å (Supplementary Figure S9).

## Conclusion

This study shows that careful evaluation of ^1^H NMR titration data from the entire molecule is required to fully understand the binding behaviour of anion-host complexes. In our case examination of the urea protons alone (as is typically reported) was unable to successfully characterise the binding stoichiometry. As demonstrated, subtle shifts in the peak position for other proton resonances provided evidence of higher order complex stoichiometries. In particular the formation of 1:2 and 2:1 complexes are apparent.

## Experimental

### Synthesis

All chemicals used in the synthesis of **4**, were obtained from Sigma-Aldrich, except ethanol which was obtained from Merck Pty. Ltd. (Victoria, Australia). NMR spectroscopy samples were dissolved in DMSO-d6 (D99.9%, Cambridge Isotope Laboratories Inc, MA, USA) unless otherwise specified. Spectra were recorded on one of a Bruker Avance III 400 or 600 spectrometers. NMR Spectra were collected at 400 MHz (^1^H) and 100 MHz (^13^C) at 26 °C, or at 600 MHz (^1^H) and 150 MHz (^13^C) at 20 °C and referenced to the centre of the residual solvent peak. Peaks are reported as singlet (s), doublet (d), triplet (t), quartet (q), doublet of triplets (dt), doublet of quartets (dq), broad singlet (bs). In addition multiplets (m) are recorded as a range of chemical shift values. Coupling constants (J) are reported in Hz.

Accurate mass analysis was performed using a Waters Synapt HDMS, with positive ion with lockspray electrospray ionisation. Spectral data was acquired in the range m/z 100 to 1000. Stock solutions of the samples were prepared at a concentration of 1 mg/mL in DMF, with working solutions prepared by dissolution in 0.5 mM sodium formate. Raffinose was used as the lock mass signal, m/z 503.1612 in negative ion mode and m/z 527.1588 for the sodium adduct in positive ion mode.

ATR-FTIR spectra were recorded on a PerkinElmer Spectrum 400 equipped with a zinc selenide (ZnSe) ATR crystal. The samples analysed were placed directly onto the crystal and appropriate force (typically around 130N) was applied to obtain the spectrum. Experiments were performed with a resolution of 4.00 cm^−1^ over a range of 4000–650 cm^−1^ with 4 scans per sample. Melting points were obtained on a Sanyo Gallenkamp Variable Heater.

#### *N*-Allyl-4-bromo-1,8-naphthalimide (**2**)

Synthesis of **2** was undertaken according to the previous method by Niu *et al*.^5^. 4-Bromo-1,8-naphthalic anhydride (2.031 g, 7.3 mmol) (**1**), was stirred in absolute ethanol (45 mL). Allylamine (591 μL, 7.9 mmol) was immediately added to the stirred solution and the solution heated under reflux. After two hours, the reaction was removed from the heat, and the product, **2**, precipitated upon standing. The product was collected using a Hirsch funnel yielding 2.18 g (94%) of **2** as a pale white powder. mp: 143.2–145.1 °C (lit^14^. 142–144 °C); ^1^H NMR (600 MHz, DMSO-d6): δ 8.61–8.58 (m, 2H) 8.36 (d, J = 7.8 Hz, 1H), 8.25 (d, J = 7.8 Hz, 1H), 8.04–8.01 (m, 1H), 5.97–5.90 (m, 1H), 5.17 (dq, J = 1.2 Hz, 15.8 Hz, 1H), 5.12 (dq, J = 1.2 Hz, 10.4 Hz, 1H) 4.65 (dt, J = 1.2 Hz, 5.2 Hz, 2H).

#### *N*-Allyl-4-(4-aminobenzylamine)-1,8-naphthalimide (**3**)

**2** (1.020 g, 3.23 mmol), was stirred at 110 °C. After ten minutes 4-aminobenzylamine (2.1 mL, 18.5 mmol) was added and stirring continued at 110 °C. After one hour, the reaction was removed from the heat and water was added to precipitate the product. The product was collected using a Hirsch Funnel and recrystallised from ethanol yielding 0.839 g (73%) of **3** as a bright yellow powder. mp: 182.5–185.4 °C; ^1^H NMR (600 MHz, DMSO-d6): δ 8.76 (d, J = 8.4 Hz, 1 H), 8.44 (d, J = 7.2 Hz, 1 H), 8.38 (t, J = 6.0 Hz, 1 H), 8.18 (d, J = 8.4 Hz, 1 H), 7.72–7.69 (m, 1 H), 7.06 (d, J = 8.4 Hz, 2 H), 6.71 (d, J = 9.0 Hz, 1 H), 6.51 (d, J = 8.4 Hz, 2 H), 5.94–5.87 (m, 1 H), 5.08–5.07 (m, 1 H), 5.06 (dq, J = 1.2 Hz, 10.4 Hz, 1 H), 4.99 (s, 2 H), 4.61 (dt, J = 1.2 Hz, 5.2 Hz, 2 H), 4.45 (d, J = 6.0 Hz, 2 H); ^13^C NMR (100 MHz, DMSO-d6): δ 163.4, 162.5, 150.6, 147.7, 134.1, 132.3, 130.7, 129.4, 128.6, 127.9, 124.9, 124.3, 121.7, 120.2, 115.9, 113.9, 107.5, 104.5, 45.8, 41.3; FTIR(ATR) (υ max solid/cm^−1^): 3387, 1683, 1673, 1634, 1615, 1575, 1542, 1515, 1449, 1417, 1391, 1365, 1333, 1287, 1241, 1172, 1107, 985, 944, 907, 820, 804, 758, 668; Accurate Mass Analysis calcd. for C_22_H_20_N_3_O_2_, 358.1500; found 358.1552; calcd. for C_22_H_19_N_3_O_2_Na, 380.1375; found, 380.1376.

#### *N*-Allyl-4-(4-(*N*-phenylureido)benzylamino)-1,8-naphthalimide (**4**)

**3** (0.203 g, 0.568 mmol), was dissolved in anhydrous DMF (6 mL) and stirred at room temperature. Phenyl isocyanate (92.6 μL, 0.852 mmol) and TEA (6 drops) were added to the stirred solution. After 24 hours, water was added to the reaction to precipitate of the product **4** which was removed by filtration as a bright yellow powder (0.254 g, 94%). mp: 221.6–223.7 °C; ^1^H NMR (600 MHz, DMSO-d6): δ 8.78 (d, J = 8.4 Hz, 1H), 8.67 (bs, 1H), 8.65 (bs, 1H), 8.49 (t, J = 6.0 Hz, 1H), 8.45 (d, J = 7.2 Hz, 1H), 8.18 (d, J = 8.4 Hz, 1H), 7.75–7.72 (m, 1H), 7.43–7.40 (m, 4H), 7.31 (d, J = 8.4 Hz, 2H), 7.27–7.24 (m, 2H), 6.96–6.94 (m, 1H), 6.70 (d, J = 8.4 Hz, 1H), 5.93–5.88 (m, 1H), 5.08–5.07 (m, 1H), 5.07–5.04 (m, 1H), 4.61–4.60 (m, 4H); ^13^C NMR (100 MHz, DMSO-d6): δ163.4, 162.5, 152.5, 150.5, 139.7, 138.6, 134.1, 133.2, 131.5, 130.8, 129.2, 128.7, 128.6, 127.5, 124.5, 121.8, 121.8, 120.3, 118.4, 118.1, 115.9, 107.9, 104.6, 45.6, 41.3; FTIR(ATR) (υ max solid/cm^−1^): 3399, 3296, 1684, 1635, 1576, 1542, 1498, 1442, 1417, 1388, 1367, 1339, 1313, 1233, 1111, 1025, 935, 835, 774, 693; Accurate Mass Analysis calcd. for C_29_H_25_N_4_O_3_, 477.1927; found, 477.1929; calcd. for C_29_H_24_N_4_O_3_Na, 499.1746; found, 499.1748.

### Binding studies

Fluorescence spectrophotometry was performed on a Varian Cary Eclipse Fluorescence Spectrophotometer (Varian Inc, Melbourne, Australia) using a 1 cm quartz cell. The excitation wavelength was positioned at the absorption maximum of 503.93 nm. Emission spectra were recorded from 510 nm–700 nm with emission and excitation slit widths of 5 mm. Solutions of **4** were prepared at 0.002 M in hydrated DMSO (0.5% v/v water in DMSO). In order to keep the concentration of host constant throughout the titration, the required anion solution was prepared by dissolving the tetrabutylammonium salt in 0.002 M **4**. Over the course of a titration up to twenty equivalents of the anion were added.

All ^1^H NMR spectroscopy titration experiments were performed on a Bruker Avance III 600 spectrometer at 600 MHz. Samples were prepared in DMSO-d6 with 0.5% water added (herein referred to as hydrated DMSO-d6) as per Caltagirone *et al*.^15^ in order to account for water absorbed from the atmosphere by the highly hygroscopic DMSO. All spectra were recorded at 20 °C. Solutions of **4** were prepared at 0.01 M. In order to keep the concentration of host constant throughout the titration, the required anion solution was prepared by dissolving the tetrabutylammonium salt in 0.01 M **4**. Over the course of a titration up to one hundred and twenty equivalents of the anion were added.

Binding constants were determined using global fitting of the ^1^H NMR data using HypNMR (http://www.hyperquad.co.uk/), with a range of different stoichiometries. A stepwise model of 1:1, 2:1 and 1:2 **4**:anion was found to best represent the experimental data.

### Data availability

Raw data will be made available upon request.

## Electronic supplementary material

Supplementary Information
