# Supplementary material for: Insights into the complexation of N-Allyl-4-(4-(N-phenylureido)benzylamino)-1,8-naphthalimide with various anions
Source: Sci Rep. 2017 May 31;7:2512. doi: 10.1038/s41598-017-02470-0 (PMC5451411; doi:10.1038/s41598-017-02470-0)
Supplement: Supplementary file 1 — Supplementary Information [file 41598_2017_2470_MOESM1_ESM.pdf]

Supporting Information: Insights into the complexation of N-Allyl-4-(4-(N-phenylureido)benzylamino)-1,8-naphthalimide with various anions

Andrew J Blok[1,2], Martin R Johnston[1,2] and Claire E Lenehan[1*]

[1]School of Chemical and Physical Sciences, Flinders University, Sturt Road, Bedford Park, South Australia, Australia

[2]Flinders Centre for NanoScale Science and Technology, Flinders University, Sturt Road, Bedford Park, South Australia, Australia

[*]claire.lenehan@flinders.edu.au

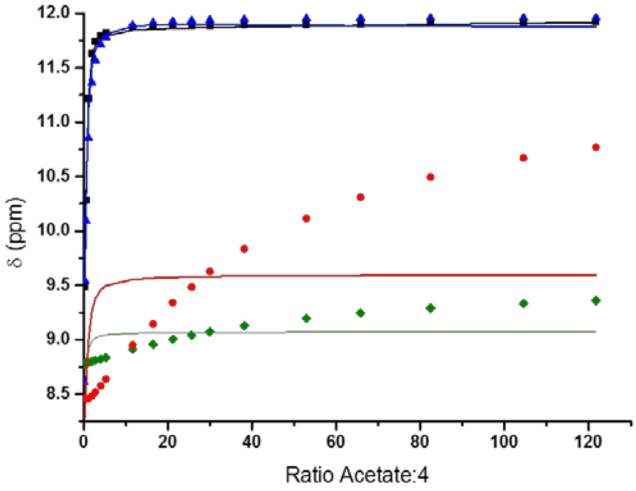

Supplementary Figure S1. ^{1}H NMR (600 MHz) binding curves for urea (observed values represented by the black square and blue circle, calculated values shown by the coloured line), C5 naphthalimide (observed values represented by the green diamond, calculated values shown by the coloured line) and 4-amino NH protons (observed values represented by the red circle, calculated values shown by the coloured line) calculated fits using a 1:1 model. As can be observed, the calculated fits are poor for the C5 and 4-amino protons.

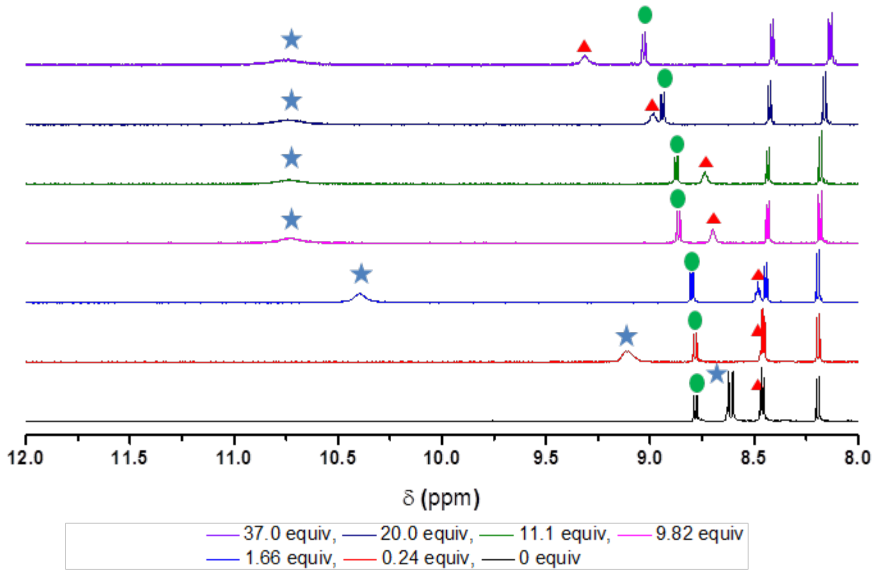

Supplementary Figure S2: ^{1}H NMR spectra (600 MHz, 6.24 mM, 293 K), of **4** complexed with tetrabutylammonium dihydrogen phosphate in hydrated (0.5% v/v) DMSO-d6. The blue star indicates the signal arising from the urea N-H protons, the green circle the signal arising for the C5 naphthalimide proton and the red triangle the 4-amino NH proton)

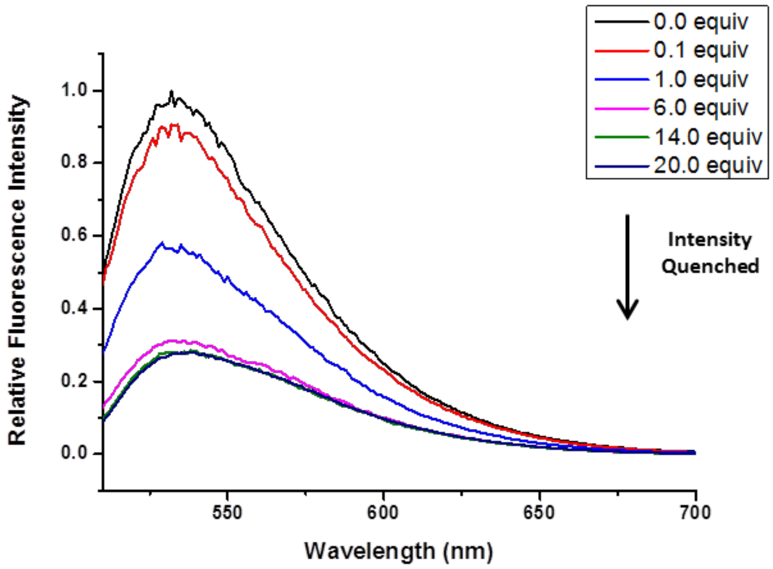

Supplementary Figure S3: Fluorescence spectra of **4** with increasing equivalents of tetrabutylammonium dihydrogen phosphate in hydrated (0.5% v/v) DMSO, λ_{ex} = 503.93 nm

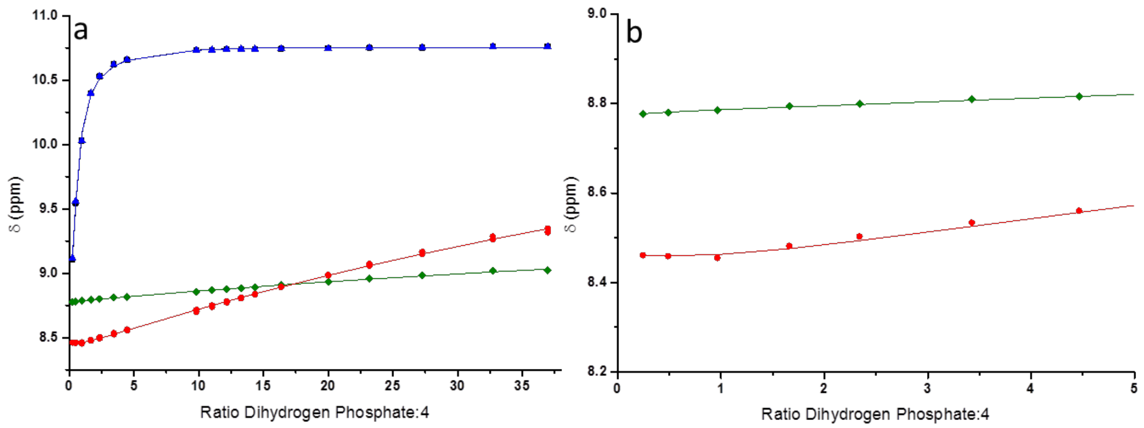

Supplementary Figure S4. (a) ^{1}H NMR (600 MHz) binding curves for urea (observed values represented by the black square and blue circle, calculated values shown by the coloured line), C5 naphthalimide (observed values represented by the green diamond, calculated values shown by the coloured line) and 4-amino NH protons (observed values represented by the red circle, calculated values shown by the coloured line) calculated fits using a 2:1, 1:1 and 2:1 binding model in HypNMR for complexes formed between **4** and tetrabutylammonnium dihydrogen phosphate. (b) An expansion of Figure S3a showing the change in chemical shift of the C5 naphthalimide and 4-amino NH protons (represented as in Figure S3a.)

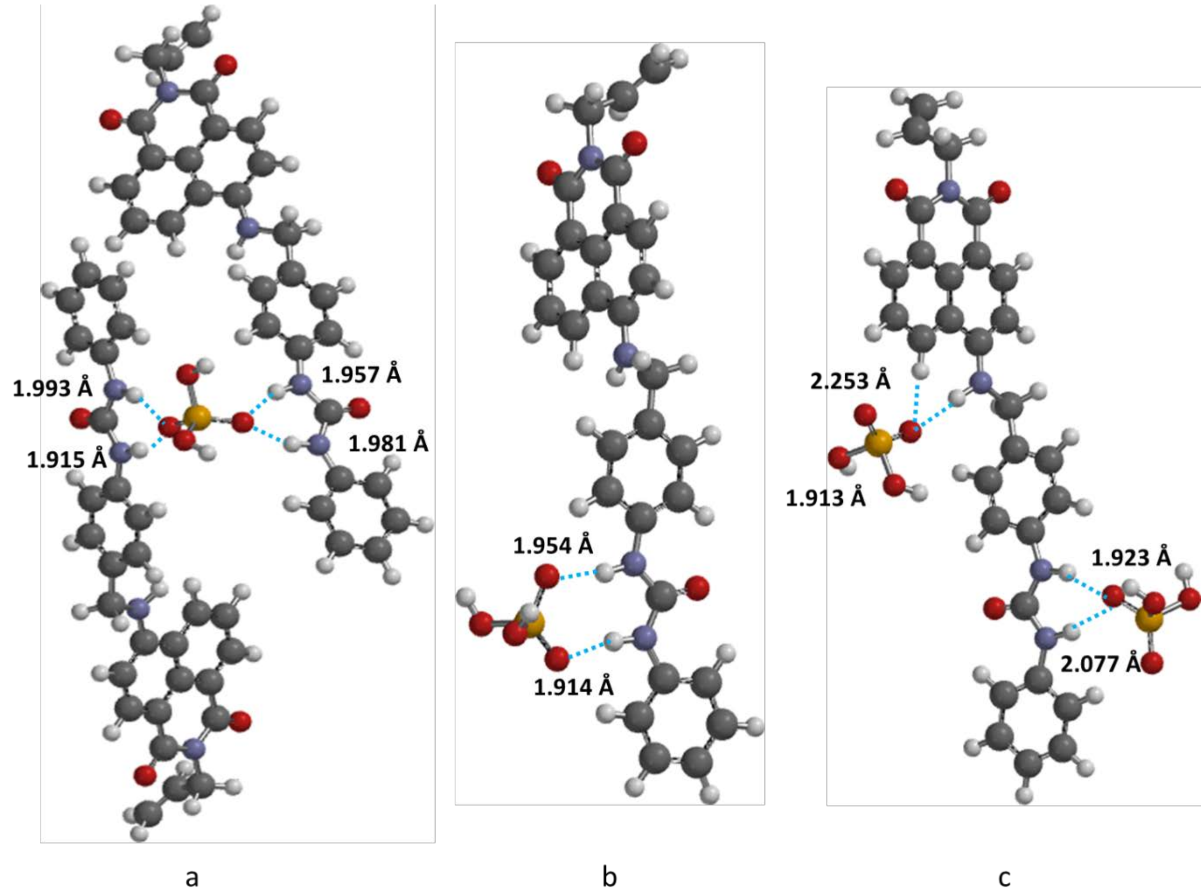

Supplementary Figure S5. Proposed structure of adducts formed between **4** and dihydrogen phosphate showing H –bonded pairs and bond distances (Hartree Fock 631G*).

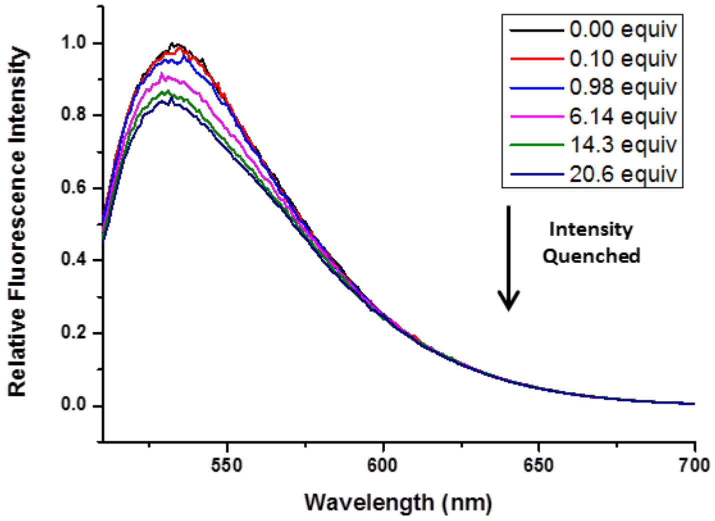

Supplementary Figure S6: Fluorescence spectra of **4** with increasing equivalents of tetrabutylammonium bromide in hydrated (0.5% v/v) DMSO, λ_{ex} = 503.93 nm

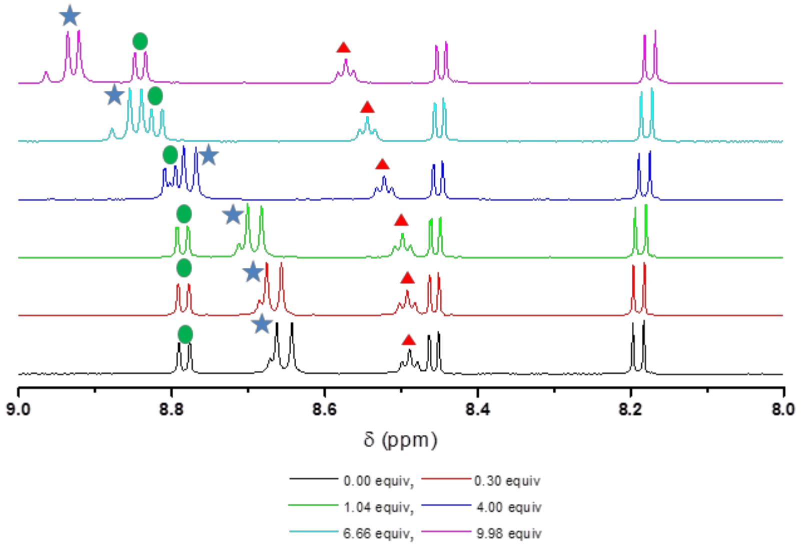

Supplementary Figure S7: ^{1}H NMR spectra (600 MHz, 6.24 mM, 293 K), of **4** complexed with tetrabutylammonium bromide in hydrated (0.5% v/v) DMSO-d6. The blue star indicates the signal arising from the urea N-H protons, the green circle the signal arising for the C5 naphthalimide proton and the red triangle the 4-amino NH proton).

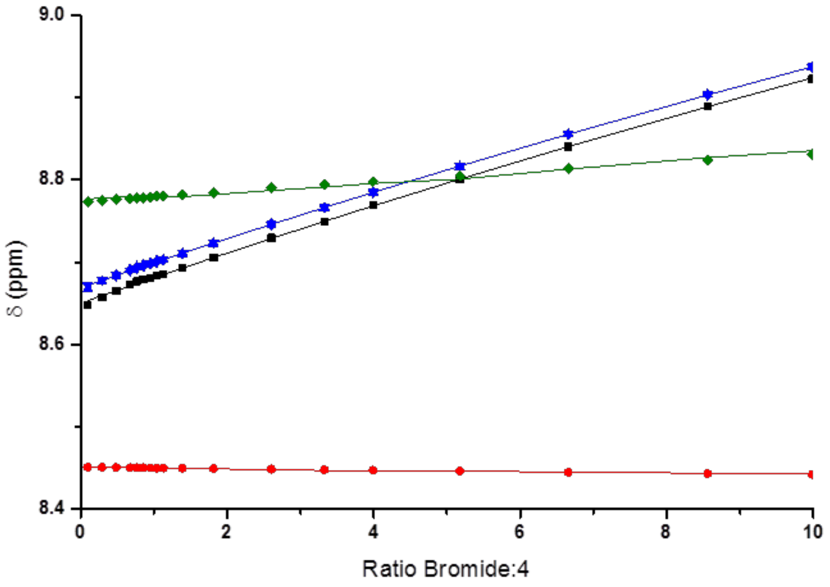

Supplementary Figure S8. ^{1}H NMR (600 MHz) binding curves for urea (observed values represented by the black square and blue circle, calculated values shown by the coloured line), C5 naphthalimide (observed values represented by the green diamond, calculated values shown by the coloured line) and 4-amino NH protons (observed values represented by the red circle, calculated values shown by the coloured line) calculated fits using a 1:1 binding model in HypNMR for complexes formed between **4** and tetrabutylammonnium bromide.

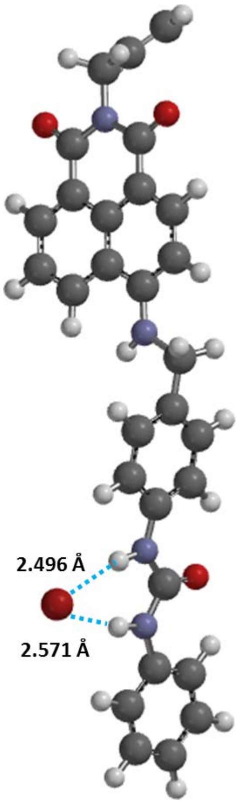

Supplementary Figure S9. Proposed structure of adducts formed between **4** and bromide showing H –bonded pairs and bond distances (Hartree Fock 631G*).
